# Notes on Certain Vegetable Poisons

**Published:** 1857-01

**Authors:** William Pickells

**Affiliations:** Cork.


### NOTES ON CERTAIN VEGETABLE POISONS.

By WILLIAM PICKELLS, M.D., Cork.

ACONITE. Some late events have a good deal roused public attention (which seemed not to have been sufficiently awake) to the dangerous qualities of the aconite (*aconitum Napellus*); particularly the lamentable occurrence during the present year in Scotland, of the deaths of three persons (two of them clergymen) in consequence of the root being plucked in mistake for, and eaten as, horseradish. The alkaloid aconitine is, according to Dr. Taylor, "a most formidable poison, exceeding all others in its effects." Dr. Headland states: "The tenth of a grain taken into the stomach would certainly kill a man." He considers it "by far the most powerful of all known poisons." The very virulent nature of the plant was well known to the ancients, by whom it was reputed to be the "quickest of all poisons." Juvenal, in several passages, alludes to the frequent use of aconite as an instrument of secret poisoning or of assassination, it being, however, never given to the poor.

"Nulla aconita bibuntur fictilibus."

"Shall such a one," he says (Sat. 1, l. 158), "who gave aconite to three uncles, look down on us (*despiciet nos*) from his vehicle decked with pensile plumes." Virgil, in the praises he lavishes, in one of his Georgics, on the natural advantages of Italy, includes its being free from the aconite;

"Nec miseros fallunt aconita legentes", implying that fatal accidents had often happened in those countries in which it grew wild, from its being plucked in mistake for, and eaten as an esculent (horseradish). Unfortunately, we cannot in these islands,—though nature has done her part, the plant not being a native,—boast a like exemption; the aconite, known by the trivial name of monkshood, being extensively cultivated in gardens for ornamental purposes; hence have happened of late years (as in the instance above mentioned) those fatal results, in consequence of the root being plucked in mistake for and, served up at table, eaten as horseradish. Nor is the cultivation of aconite in gardens limited to those of the middle and affluent clssses. In this part of Ireland, but still more, I understand, in the North, it is, in numerous instances, cultivated in the gardens of small cottagers, who appear to be little aware of the danger. Children are fond of sucking the flower, tempted by the honey in the nectarium. In the last number of the *Lancet* (July 26th) is the report by Dr. Massey, of Nottingham, of the case of a small cottager, who died from having eaten, with his usual supper, a small portion of the root of the monkshood, which grew in his garden. In a former number of the *Lancet* was given the case of the child of a small cottager, living at Islesworth, whose death was occasioned by having eaten of the leaves of the monkshood which grew in the garden.*

The shores of the Black Sea were, it appears, in former times (if the stigma do not still apply), much infested by the frequency of aconite. Ovid has

"Scytheis aconiton ab oris."

The Black Sea was sometimes called the "Scythian Sea". Pliny stigmatises the port "Acone,"—200 miles from Pontus, on the eastern shore of the Black Sea, as dire (*dirus*) from the poison aconite. In the list of poisonous plants known to Dioscorides, given by Dr. Osborne, in his paper, in the *Dublin Medical Journal*, vol. xv, on the state poison of the Athenians, occurs "aconite and the honey of Heraclea in Pontus, where that plant abounds"; imparting, it would hence appear, to the honey of the place, a poisonous quality from bees feeding on the flowers.

The author of a much read popular publication of the present day (*Household Words*), attributes the poisonous quality of the Pontic honey to the abundance in the country of the rhodo-

* The melancholy death of the paymaster at Navan, near Dublin, in the early part of the present year (which excited so considerable a sensation), in consequence of the mistake at the apothecary's of tincture of aconite for tincture of chiretta, should serve as an effectual warning against entrusting implicitly the making up of medical prescriptions to tyros.

dendron. It is more probable, however, that it was owing to the abundance of the aconite. The Romans would not receive honey in tribute from the people of Pontus, but took from them, instead of it, a double quantity of wax. The abundance of poisons in Pontus, the present Natolia, is alluded to by Virgil in the eighth Eclogue.

> " Has herbas, atque hæc Ponto mihi lecta venena,
> Ipse dedit Mæris; nascuntur plurima Ponto."*

Aconite was probably the prime agent in those poisonings, which were formerly so common in the East; to the utter disregard, but too often in profligate pursuit of power, of the nearest ties of consanguinity,—the murderer, however, usually paying the forfeit of his guilt, in perishing by like treachery. "In Parthia, to have kings parricides was," according to Justin, "an almost established custom (*quasi solenne erat*)". "Phraates, not content with a share of the government, determined on the murder of his father (Orodes), and took an opportunity of administering aconite to him; but the king being afflicted with dropsy, the poison working on the disease removed the dropsical matter, and Orodes began to recover, when the diabolical son, impatient to reign without control, stifled him with his pillow!" (Lindsay's *Parthian History and Coinage,* from Plutarch, *in Crasso.*) Orodes it was who rendered the Parthian name so terrible to the Romans, by having destroyed Crassus with the whole Roman army! "Phraates, after putting to death his father, put to death" (using, probably, aconite as an instrument) "fifty brothers, sons whom his father had by different concubines; and still not stopping with sons, as he saw that the nobles, from his continual crimes, were adverse to him, he put to death every adult son who might be nominated king. In the sequel, he was himself put to death by his own son."† In the case of Alexander the Great, who died of poison in Babylon, the poison administered was, we are told by the historian, "so strong, that it could not be contained in brass, nor iron, nor shell, nor otherwise carried than in a horse's hoof."

* So constantly apprehensive was Mithridates, King of Pontus, of being poisoned, that he had recourse to an antidote composed of fifty ingredients, by the daily use of which he was said to have become poison-proof. His multifarious yet nugatory farrago retained a place, under the name of Mithridate, in the Pharmacopœia, up to nearly the end of the last century. Sinope, still retaining its ancient name, the scene of a barbarous massacre of the Turks by the Russians during the late war on the shores of the Black Sea, was the capital of Pontus in the time of Mithridates.

† While reading in the pages of Justin (from whom is the above), particularly as regards the East, the crimes perpetrated in the pursuit of power, a doubt forces itself on the mind, whether we are reading the annals of what actually happened in this world, or " the records of hell."

"Stygium" was an epithet sometimes applied to the aconite, to denote its intensity as a poison. The water of the Styx, it was said, was so poisonous, or corrosive, that it could be kept only in a horse's hoof! Hence, laying aside hyperbole, the poison given to Alexander was probably aconite. It was given to him by his cup-bearer, who had been suborned by Antipater. "Philippus et Iollas prægustare ac temperare potum regis soliti, in aquâ frigidâ venenum habuerunt, quam prægustatæ jam potioni supermiserunt."

TOXICUM. What was the nature of the poison, strictly so called by the ancients, whence our term *Toxicology*, applied to the study of poisons in general,—a study which, raised by the labours of Orfila to the rank of a science, has become so useful as subserving the ends of justice in questions of medical jurisprudence? "Venenum quoddam," says the scholiast, "dicitur Toxicon, quod Dioscorides scribit ita nominari videri ex eo, quod barbarorum sagittæ eo illinerentur." But the same scholiast adduces other reasons for the name, scilicet: "τοξικον, nominatum esse φαρμακον illud δια το ὁμοιως τοις τοξευμασιν ἀναιρειν παραχρημα." This etymology appears to agree with that given by the author of the article *Toxicology*, in the *Dictionary of Practical Medicine*: "from Τοξικον, venenum, itself derived from τοξον, a bow or arrow, by metonymy, a poison." Another etymology (drawn from fabulous history) was "Quia ex του αἱματος της ὑδρας ἀνεφυη ἡν τοξοις ἀνειλεν ὁ Ἡρακλης." These different etymologies refer, it may be perceived, to the energy of the poison, or to the purpose to which it was applied, but give no insight into its real or specific nature. Pliny, xvi, 10, speaking of the yew tree, "de taxo arbore," says: "Sunt qui et taxica, hinc appellata venena, quæ nunc toxica dicimus, quibus sagittæ tinguntur"; implying, that the poison with which arrows (the points) were poisoned was taken from the yew. Ainsworth has, "Toxicum [qu. taxicum, e taxo arbore, *vide* Pliny, xvi, 10: Potius a Gr. τοξικον], poison."* Under "Taxeus" he quotes Florus, as saying of a certain poison, "quod venenum ex taxeis arboribus exprimitur." Cæsar informs us that Cativolcus, king of half of a people of Belgic Gaul, failing in an insurrection against the Romans, committed suicide with the yew, "exanimavit se taxo," that is, interprets the annotator, "with the juice of the yew." An ancient poet calls the yew lethal:

* The analogy of τοξον, the Greek name for a bow, to taxus, the tree of which the best bows were made, is suggestive that arrows too were made from it. Shakespeare says, "Bows of double fatal yew."

"Lethali taxo, et Scythicis medicata venenis."

The custom of the barbarians poisoning their arrows with toxicum, is alluded to by Ovid in one of his epistles from Tomi, the place of his exile, not far from the mouths of the Danube:—

"Aspicis et mitti sub adunco toxica ferro,
Et telum causas mortis habere duas."

In modern times, the poisonous qualities of the yew have been doubted by some, and even denied. In the *Treatise on Poisons* of Christison, yew poison is omitted. Sir James Smyth, in his *Flora Britannica*, speaking of the yew, says: "The leaves are fœtid and very poisonous, and prove speedily fatal to cattle, accidentally tasting them, when young and tender. The berries are not poisonous." The fatal effects of the yew on cattle were noticed so far back as the time of Dioscorides. Lindley says, "The leaves of the common yew are fœtid, and very poisonous, especially to horses and cows. The berries are not dangerous." Taylor, in his work on *Medical Jurisprudence*, gives, under the title "Yew Poison," an instance of three children who died from a tablespoonful of the fresh leaves, administered as a vermifuge; "showing," as he observes, "the fallacy of the vulgar, but erroneous notion, that the yew leaves are not poisonous when fresh, and that, in any case, they act only mechanically." He gives also the cases of three children who died from having eaten the berries; "proving," he observes, "that there is a specific poison in the yew, since it exists in the berries."

Should further inquiry confirm the supposition, that the poison known to the ancients by, strictly, the name of toxicum, was no other than yew poison, the curious fact would be revealed, that a poison, deemed in our times so doubtful as such, or insignificant, as to be omitted in the works of Christison, has given a name to the science which treats of all poisons, vegetable, animal, or mineral.*

---

* The term *intoxication*, applied to a vice, which is unfortunately the bane of some countries, is derived by Johnson from "in" and "toxicum", by Webster from "in" and "toxicum"; "which Pliny informs us is from taxus, a species of tree, in Greek σμιλαξ." Might the juice of the yew have been somehow taken advantage of for a stimulant effect of this sort, thus adding another to the various devices of man in different countries for temporarily depriving himself of the use of reason? The word "intoxicatio" does not occur in Ainsworth, the over-drinking of the nations of the South being in wine. Hence it would appear that ebriety produced by the toxicum (if the toxicum did supply a stimulant of this sort) was confined to some northern latitudes, in which the vine did not grow. The drunkenness of the Scythian nations was proverbial.

